# A global daily soil moisture dataset derived from Chinese FengYun Microwave Radiation Imager (MWRI)(2010–2019)

**DOI:** 10.1038/s41597-023-02007-3

**Published:** 2023-03-14

**Authors:** Panpan Yao, Hui Lu, Tianjie Zhao, Shengli Wu, Zhiqing Peng, Michael H. Cosh, Li Jia, Kun Yang, Peng Zhang, Jiancheng Shi

**Affiliations:** 1 State Key Laboratory of Remote Sensing Science, Aerospace Information Research Institute, Chinese Academy of Sciences, Beijing, 100101 China; 2 Ministry of Education Key Laboratory for Earth System Modeling, Department of Earth System Science, Tsinghua University, Beijing, 100084 China; 3 National Satellite Meteorological Center, China Meteorological Administration, Beijing, 100081 China; 4 Hydrology and Remote Sensing Laboratory (HRSL), United States Department of Agriculture-Agricultural Research Service (USDA-ARS), Beltsville, MD 20705 USA; 5 National Space Science Center, Chinese Academy of Sciences, Beijing, 100190 China

**Keywords:** Hydrology, Hydrology

## Abstract

Surface soil moisture (SSM) is an important variable in drought monitoring, floods predicting, weather forecasting, etc. and plays a critical role in water and heat exchanges between land and atmosphere. SSM products from L-band observations, such as the Soil Moisture Active Passive (SMAP) Mission, have proven to be optimal global estimations. Although X-band has a lower sensitivity to soil moisture than that of L-band, Chinese FengYun-3 series satellites (FY-3A/B/C/D) have provided sustainable and daily multiple SSM products from X-band since 2008. This research developed a new global SSM product (NNsm-FY) from FY-3B MWRI from 2010 to 2019, transferred high accuracy of SMAP L-band to FY-3B X-band. The NNsm-FY shows good agreement with in-situ observations and SMAP product and has a higher accuracy than that of official FY-3B product. With this new dataset, Chinese FY-3 satellites may play a larger role and provide opportunities of sustainable and longer-term soil moisture data record for hydrological study.

## Background & Summary

Surface soil moisture (SSM) is essential for agriculture, ecosystem, weather, climate system and human health^1–4^. SSM is a boundary condition, affecting the evapotranspiration and infiltration in water cycle, as well as the latent and sensible heat fluxes in land surface energy balance^5–9^. Soil moisture is necessary for crop growth and yield, and soil moisture plays important role in monitoring disaster (e.g., drought, flood, landslide etc.)^10–12^ and climate extremes (e.g., heatwave)^13,14^. Soil information can help improve weather forecasting accuracy and model performance when using soil moisture observed by satellites into land surface model^15–17^. Long-term and spatio-temporally consistent SSM datasets are therefore necessary for those applications and scientific researches^18,19^.

Microwave remote sensing has proven successful for providing spatial and temporal distribution of global soil moisture from satellite missions, especially L band passive sensors in recent years. C/X/K band sensors (SSM/I (Special Sensor Microwave/Imager), AMSR-E/AMSR2 (Advanced Microwave Scanning Radiometer), MWRI etc.^20,21^) provide 40 years SSM observations at sensing depth of ~1 cm, with more uncertainties due to vegetation effects compared with L band. In contrast, L band radiometers (SMOS (Soil Moisture and Ocean Salinity) and SMAP) provide only 14 years and 8 years SSM products with higher accuracy at sensing depth of ~5 cm, with more penetration into vegetation and less opaque of atmosphere^22,23^.

To meet more application requirements for long-term datasets, there are generally two strategies: 1) The first strategy is SSM retrieval with one consistent algorithm, which requires inter-calibrated microwave observations. Different methods have been explored, including the regression method^24^, the neural network (NN) method^25,26^, the land parameter retrieval model (LPRM), and the recent multi-channel collaborative algorithm (MCCA)^27^, etc. 2) The second strategy is to blend multi-satellite products. Owe^28^ developed a dataset dating back to 2007 by applying LPRM to the entire brightness temperature (TB) observed by C- and X-bands sensors. Then, Liu^29,30^ combined products from active and passive microwave sensors by rescaling active and passive products to a reference land surface model data using a cumulative distribution function (CDF) matching approach. On the basis of above works, Gruber^31^ proposed a triple collocation analysis (TCA)-based method for merging soil moisture taking into account the error characteristics of the individual active and passive datasets, forming the basis of Climate Change Initiative soil moisture (CCIsm) product version v03.x and higher. SMOPS^32^ (soil moisture operational product system) provides a daily global SSM product with high spatial coverage (2017.03-present) that merged soil moisture retrievals from multiple satellites.

CCIsm^33–36^ is a publicly available and widely used long-term soil moisture dataset. While CCIsm has sufficient record length (from 1978.11 to present) for climatological studies, it depends on numerous sensor specifications and CDF matching reduces inter-annual variability and climatological trends. It is found that the accuracy of CCIsm depends on regions and seasons, and the accuracy could be further improved through blending more SSM products. To be noted, the CCIsm only blends the FY-3B observations from June 2011 to August 2019. We developed a NN method extending L band (SMOS or SMAP) benefits to previous C/X band AMSR-E/AMSR2 data, and then released a nearly 20 years long-term soil moisture product^37,38^ (NNsm-AMSR), with high accuracy similar to SMAP but greater spatial coverage than SMOS and SMAP. Unfortunately, NNsm-AMSR has a temporal gap of 9 months due to data gaps between AMSR-E and AMSR2 sensors, which limits its applications. On the other hand, MWRI onboard Chinese FY-3 satellites has almost the same frequencies configuration with AMSR-E/AMSR2 except C-band, and the FY-3 (B/C/D) data are available since 2010. The development of FY-3 SSM products can be an important input for future blended SSM products.

**Fig. 1:**
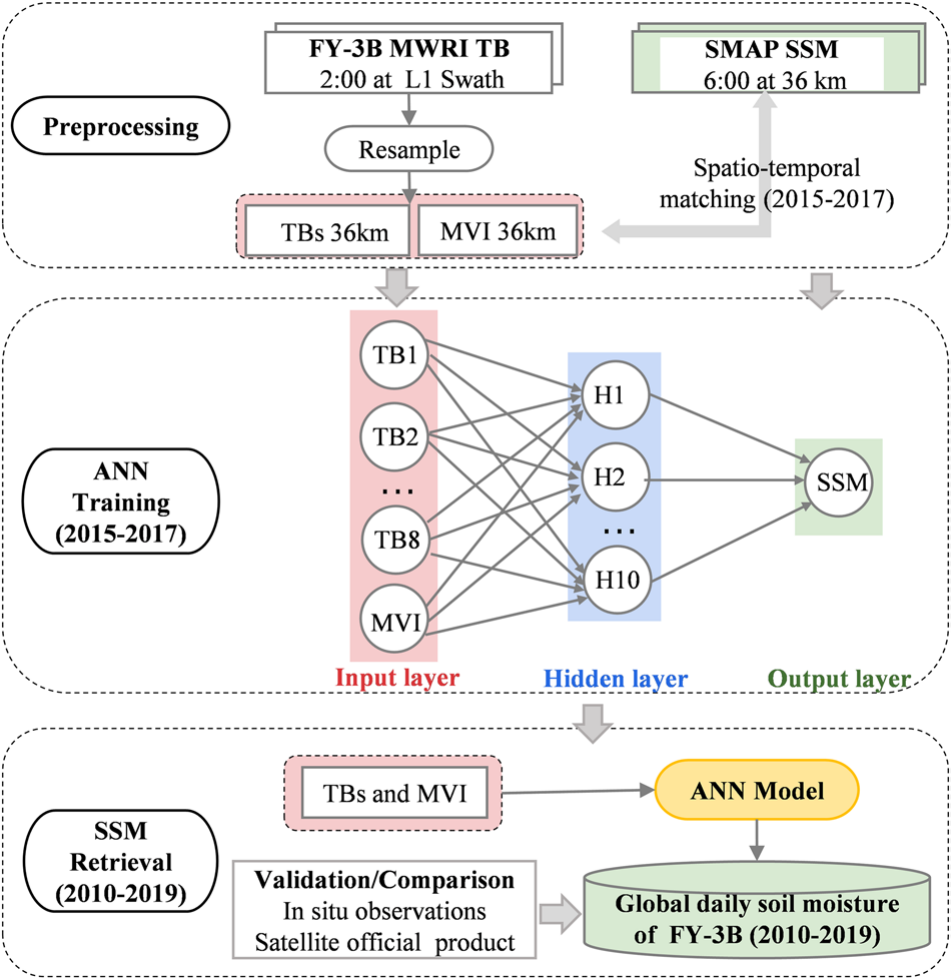
Flow chart of soil moisture retrieval of NNsm-FY dataset.

This research developed an SSM product (named as NNsm-FY) at 36 km resolution from 2010 to 2019 by transfering high accuracy of SMAP L-band SSM to X-band MWRI of FY-3B. Figure 1 shows the scheme of the NNsm-FY dataset development. Validation against in situ data from representative networks demonstrate that the NNsm-FY matches well with in situ SSM, with similar accuracy to SMAP (~0.04 m^3^/m^3^). This dataset provides a new soil moisture product of Chinese FY-3 with high accuracy, and fills in the gap (2011.11–2012.06) between the effective end of AMSR-E and beginning of the AMSR2, such as a previously developed long term dataset NNsm-AMSR^39^, and intends to provide opportunities for sustainable and longer-term soil moisture for hydrological or climate change study at global or regional scale.

## Methods

In our research, we used two kinds of data. SMAP level 3 soil moisture product (SMAPL3sm, Version 6), available at website of National Snow and Ice Data Center (NSIDC, https://nsidc.org/data/smap), are in a global cylindrical 36 km Equal-Area Scalable Earth Grid, Version 2.0 (EASE-Grid 2.0), with size of 406 rows × 964 columns. TBs of FY-3B MWRI are re-calibration level 1 swath data, provided by National Satellite Meteorological Center (NSMC). And the footprint sizes of MWRI 10.65 GHz, 18.7 GHz, 23.8 GHz, 36.5 GHz, 89 GHz are 51 km × 58 km, 30 km × 50 km, 27 km × 45 km, 18 km × 30 km and 18 km × 30 km. Table 1 summarizes the data used, their spatial resolutions, and provenance.

**Table 1:** Details of data used in the study.

Data	Source	Time Period	Spatio-temporal resolution
FY-3B MWRI L1 TB	provided by NSMC	Training: 2015–2017	Swath
SMAP L3 soil moisture	https://nsidc.org/data/smap	Training: 2015–2017	36 km, Daily
FY-3B MWRI L1 TB	provided by NSMC	Retrieval: 2010–2019	Swath

A soil moisture retrieval model was developed based on artificial neural network (ANN), which is a modified version of method developed by Yao^37,39^. As shown in Fig. 1, the NNsm-FY dataset was generated with three steps:(1) data pre-processing and data matching, (2) training of soil moisture ANN model, (3) retrieval and validation of NNsm-FY dataset.

### Data Pre-processing

To match FY-3B TBs with SMAPL3sm, FY-3B TBs are firstly resampled to 36 km. We use the “drop in the bucket” method to resample FY-3B L1 swath data to 36 km EASE-Grid format. All swath data that fall within a 36 km grid cell are averaged together. Microwave vegetation index(MVI), proposed by Shi^40^, is an indicator of effect of vegetation in soil moisture retrieval. MVI is calculated by TBs of two bands, and the formula is as follows:

1 $${\rm{MVI}}\left({{\rm{f}}}_{1},{{\rm{f}}}_{2}\right)=\frac{{{\rm{TB}}}_{v}\left({{\rm{f}}}_{2}\right)-{{\rm{TB}}}_{h}\left({{\rm{f}}}_{2}\right)}{{{\rm{TB}}}_{v}\left({{\rm{f}}}_{1}\right)-{{\rm{TB}}}_{h}\left({{\rm{f}}}_{1}\right)}$$

### Training of soil moisture ANN model

The training is implemented in Matlab R2018b version. Cascade-forward neural network is selected as the training model, with “hiddenSizes” being 10, “Training function” being “trainlm (Levenberg-Marquardt)”. Mean squared error (mse) is used to evaluate the performance of the neural network. In the training period from 2015 to 2017, the training target is SMAPL3sm, and the Input data are matched FY-3 TBs from 10 GHz to 36.5 GHz and MVI derived from FY-3 TBs. The training dataset doesn’t include the frozen condition, cause the SMAPL3sm has no value when soil is frozen.

The ANN model is trained to learn the relationship between the input TBs related variables and the target SMAPL3sm. The model is trained separately for each grid cell in Matlab R2018b version. To reduce the impact of temperature in soil moisture model, FY-3 TBs at 2:00 o’clock and SMAPL3sm at 06:00 o’clock are selected. From 2015 to 2017, spatial-temporal matched FY-3B data and SMAP data form the training dataset.

Matlab randomly divide the training dataset into 3 kinds of samples: 70% training samples, 15% validation samples and 15% testing samples. The network is trained and adjusted according to its error of multiple train epochs with training samples. The validation samples are used to halt training when the network stops improving. The test sample provide an independent measure of network performance during and after training.

### Retrieval of NNsm-FY for each grid cell

After establishing the ANN relationship model between the input and the target for each grid cell, the model is applied with inputting pre-processed FY-3B TBs and MVI at the corresponding 36 km grid cell from 2010 to 2019. The retrieval is implemented using one year’s daily data over one grid cell, and the global daily soil moisture from 2010 to 2019 are obtained finally.

## Data Records

The data records^41^ contain global daily soil moisture data with a spatial resolution of 36 km, in unit of m^3^/m^3^, from November 2010 to July 2019. These data are stored in NetCDF format with one file per day, defined by two dimensions (lat, lon, respectively representing latitude and longitude) and a variable soil moisture (soil_moisture). The file name is “NNsm-FY-yyyyddd.nc”, where “yyyy” stands for year and “ddd” stands for Julian date. For example, “2019001.nc” contains the global soil moisture distribution on the first day of 2019. This dataset is freely available from National Tibetan Plateau Data Center (TPDC, http://data.tpdc.ac.cn/).

Naming convention:

NNsm-FY-yyyyddd.nc

Variable: soil_moisture = volumetric soil moisture [m^3^/m^3^]

## Technical Validation

Validation is critical for providing accurate product before widely usage. The NNsm-FY dataset was validated at different spatio-temporal scales. Before validation, the training results were evaluated by comparing with training target. The NNsm-FY dataset was firstly validated using in-situ observations. Then the performance of NNsm-FY was compared with that of FY-3B official soil moisture product (FY-sm) and ERA5 soil moisture. We previously developed a soil moisture product called NNsm_AMSR (2002–2011,2012–2020) using similar method with SMAPL3sm and AMSR-E/AMSR2 TB. Finally, the homogeneity of NNsm-FY and NNsm_AMSR was verified. For evaluation against in situ observations and other SSM products, we used the correlation coefficient (CC), root mean square error (RMSE), bias, unbiased RMSE (ubRMSE) for statistical results.

### Evaluation of training results

**Fig. 2:**
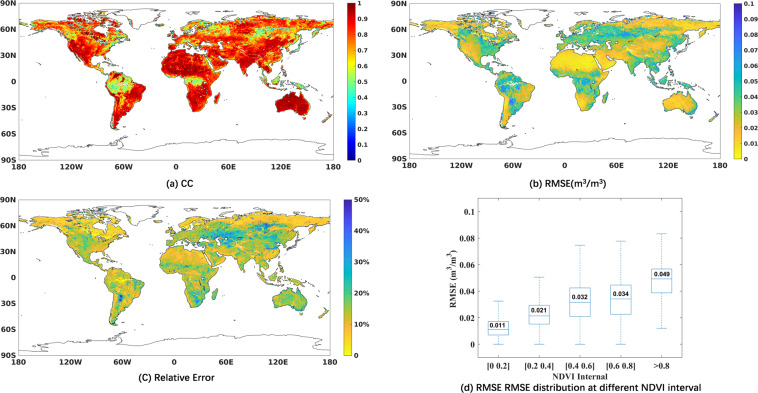
Statistical map between training result and SMAPL3sm over training period (2015–2017): (**a**) CC, (**b**) RMSE(m^3^/m^3^), (**c**) Relative error and (**d**) RMSE distribution at different NDVI interval.

To demonstrate the reliable of soil moisture ANN model, the relationship between the training results with training target SMAPL3sm is shown in Fig. 2, in terms of CC, RMSE and relative error at each grid cell over the training period. Traning results has high CC (>0.8) with the target SMAPL3sm globally, except for regions of equatorial rainforest and forest at high latitude such as part of Russia. Statistically, 57 percent of RMSEs over global land are below 0.03 m^3^/m^3^, and 29 percent of RMSEs is between 0.03 m^3^/m^3^ and 0.05 m^3^/m^3^. The high CC and low RMSE indicate that the input and the target of the model has a good correlation, and the trained result has a good agreement with the target.

The map of relative error in Fig. 2c shows a different information of error. Statistically, over global land, only 30 percent of relative error is below 10%, 54 percent of relative error is between 10% and 20%. At forest area and tundra area, where the soil moisture is generally high, the relative error is small. At the central part of Eurasia, where the soil moisture is generally low, the relative error is larger. Vegetation effect is an important influence factors in soil moisture retrieval. To test the effect of vegetation on the error level of NNsm-FY, the RMSE distribution between the training result NNsm-FY and the reference target SMAPsm is displayed in Fig. 2d, at different NDVI interval. As a whole the RMSE increases as the NDVI increases. When NDVI is between 0.6 and 0.8, the median of RMSE is 0.034 m^3^/m^3^, and when NDVI is greater than 0.8, the median of RMSE is 0.049 m^3^/m^3^.

### Validation using in situ observations

An ideal in situ validation network should has multiple sampling sites and represent the “truth” within a spatial domain that matching a satellite product grid. We choose both classical “dense” in situ networks with multiple sampling sites for validation, and “sparse” in situ networks with only one or very few sampling sites. These validation networks distribute in different climate regime and land cover in five continents.

**Fig. 3:**
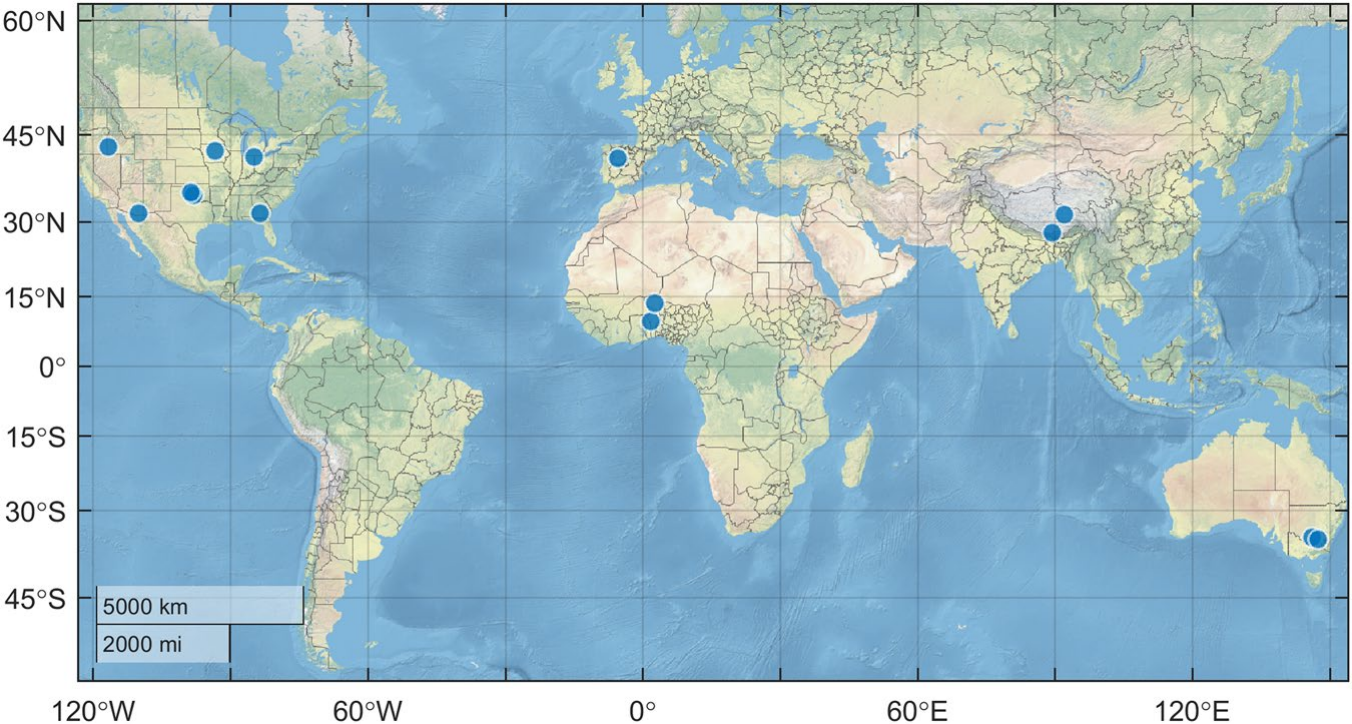
Location of in situ soil moisture validation dense networks.

1. Dense validation networks
  14 representative in situ dense networks are used for validation, shown in Fig. 3 and Table 2, including: (a) 7 United States Department of Agriculture (USDA) watershed networks^42,43^, (b) 2 Tibetan Plateau networks, (c) 2 Australian Moisture Monitoring Network (OZNet) networks^44^, (d) the REMEDHUS network, and (e) 2 African Monsoon Multidisciplinary Analysis (AMMA) networks^45^. Data of OZNet, REMEDHUS and AMMA sites are provided by International Soil Moisture Network (ISMN) (https://ismn.geo.tuwien.ac.at/) website^46,47^. As space is limited, time series of bold-face networks are shown in the following figures.

**Table 2:** List of validation dense networks.

	Location	Network Name	Sampling sites amount	Climate regime^a^	IGBP^b^ Land cover
1	USDA (North America)	Walnut Gulch	29	Arid	Shrub open
2		Little Washita	20	Temperate	Grasslands
3		Fort Cobb	15	Temperate	Croplands
4		Little River	28	Temperate	Cropland/natural mosaic
5		Saint Joseph’s	15	Cold	Croplands
6		South Fork	20	Cold	Croplands
7		Reynolds Creek	20	Arid	Grasslands
8	Tibetan Plateau (Asia)	Pali	20	Arid	Barren/Grasslands
9		Naqu	58	Polar	Grasslands
10	OZNET (Australia)	Yanco	24	Semi-arid	Croplands/Grasslands
11		Kyeamba	8	Temperate	Croplands
12	REMEDHUS (Europe)	REMEDHUS	24	Temperate	Croplands
13	AMMA (Africa)	Benin	4	Arid	Savannas
14		Niger	3	Arid	Grasslands

**Fig. 4:**
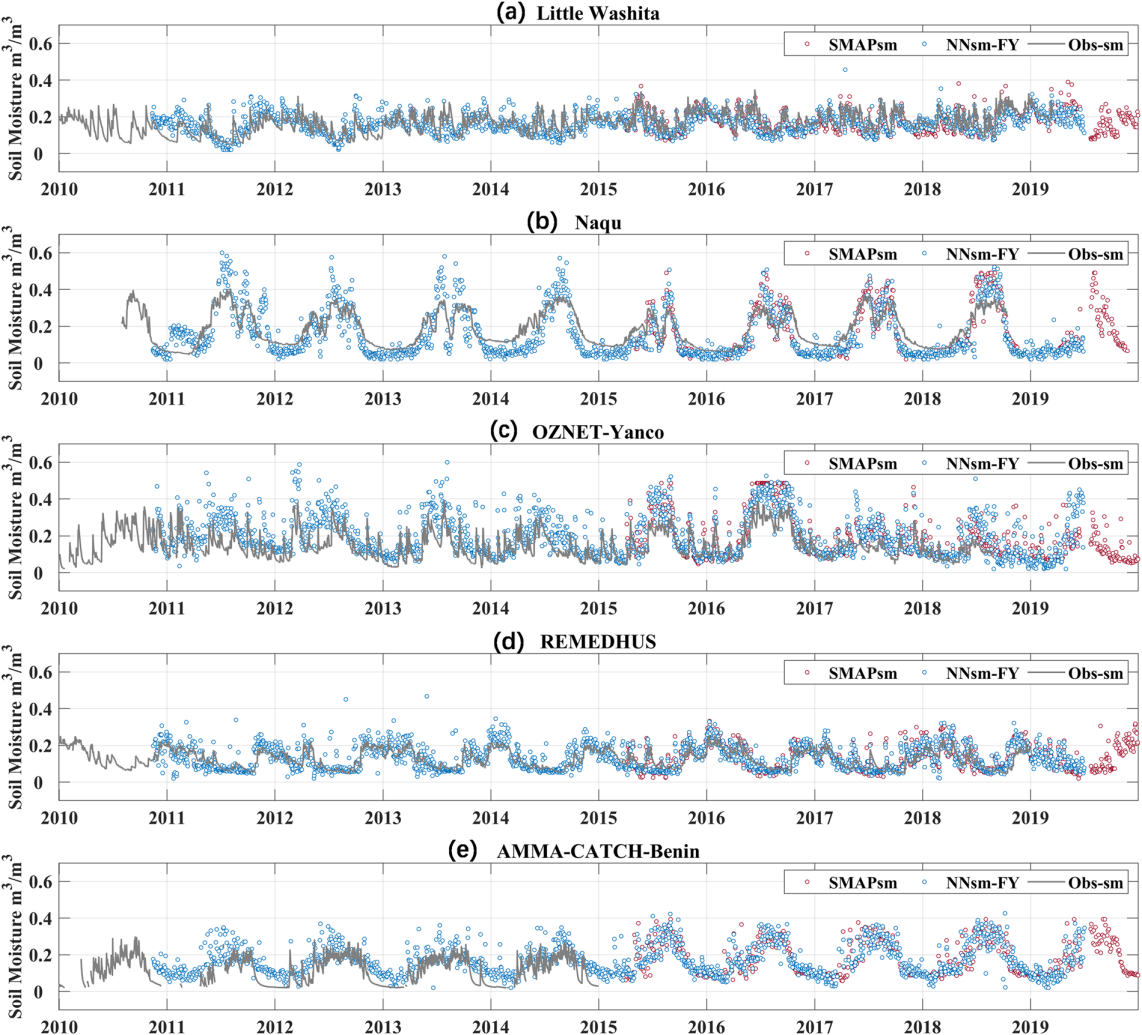
Time series comparison of the SMAPsm (red dots), NNsm-FY (blue dots) and in situ soil moisture observations (obs-sm in gray lines) for period 2010 to 2019 over in situ networks: (**a**) Little Washita, (**b**) Naqu, (**c**) Yanco, (**d**) REMEDHUS, (**e**) Benin.

Time series for bold-face networks are shown in Fig. 4 and Fig. 11.

^a^Koeppen-Geiger climate classification^58^.

^b^International Geosphere-Biosphere Program.
2. Flux sites

FLUXNET (https://fluxnet.org/) is a global network of micrometeorological tower sites that measure the carbon dioxide, water vapor, and energy between terrestrial ecosystems and the atmosphere. We use 5 flux datasets: (1) FLUXNET2015^48^, the most recent FLUXNET data product, (2) ICOS2020^49^, Warm Winter 2020 ecosystem eddy covariance flux product, (3) ICOSETC2022^50^, (4) AmeriFlux Network (https://ameriflux.lbl.gov/), and (5) TERN, the Terrestrial Ecosystem Research Network(https://portal.tern.org.au/). The half-hour soil moisture observations at 5 cm depth from those sites are used shown in Table 3.

**Table 3:** List of flux sites.

	Source	Location	Number of sites^a^	Time period^b^
1	FLUXNET2015	Global	152	1996–2014
2	ICOS2020	Europe	66	1996–2020
3	ICOSETC2022	Europe	37	2011–2021
4	AmeriFlux FLUXNET	America	44	1994–2021
5	TERN	Australia	22	2002–2022
Merger	—	Global	258	1994–2022

^a^ and ^b^: Sites which have half-hour soil moisture observations.

The performance of NNsm-FY over in situ dense networks and Flux sites are shown in time series in Fig. 4 and in a statistical result for an independent validation period (2018–2019) in Table 4.

**Table 4:** Statistical comparisons of NNsm-FY derived from FY-3B and SMAPL3sm against in situ soil moisture at 12 dense validation networks and flux sites, at an independent validation period (2018–2019), for both actual SSM and SSM anomalies.

2018–2019	NNsm-FY vs. in situ				SMAPsm vs. in situ			
	CC	RMSE	Bias	ubRMSE	CC	RMSE	Bias	ubRMSE
1.Walnut Gulch	0.71	0.029	−0.001	0.029	0.80	0.030	0.004	0.030
2.Little Washita	0.73	0.038	−0.011	0.037	0.88	0.034	−0.019	0.028
3.Fort Cobb	0.83	0.038	−0.008	0.037	0.86	0.047	−0.024	0.041
4.Little River	0.58	0.079	0.069	0.038	0.68	0.063	0.051	0.037
5.Saint Joseph’s	0.56	0.071	0.048	0.052	0.69	0.074	0.052	0.052
6.South Fork	0.35	0.063	0.019	0.060	0.57	0.068	0.022	0.065
7.Reynolds Creek	0.64	0.055	−0.047	0.027	0.77	0.046	−0.041	0.021
8.Pali	0.70	0.056	−0.052	0.023	0.75	0.052	−0.047	0.022
9.Naqu	0.91	0.099	0.014	0.098	0.94	0.091	0.028	0.087
10.Yanco	0.57	0.080	0.045	0.066	0.50	0.062	0.035	0.051
11.Kyeamba	0.59	0.103	0.033	0.097	0.57	0.093	0.032	0.087
12.REMEDHUS	0.68	0.057	0.025	0.052	0.80	0.047	0.020	0.042
13.Benin(2010–2014)	—	—	—	—	—	—	—	—
14.Niger(2010–2014)	—	—	—	—	—	—	—	—
Median	0.66	0.060	0.017	0.045	0.76	0.057	0.021	0.042
Flux Sites(Median)	0.44	0.117	0.012	0.065	0.55	0.110	0.001	0.064
**2018–2019**	**NNsm-FY vs. in situ (Anomalies)**				**SMAPsm vs. in situ (Anomalies)**			
1.Walnut Gulch	0.68	0.024	0.001	0.024	0.72	0.024	0.001	0.024
2.Little Washita	0.63	0.040	−0.013	0.038	0.82	0.040	−0.027	0.029
3.Fort Cobb	0.73	0.038	0	0.038	0.77	0.045	−0.015	0.042
4.Little River	0.72	0.037	−0.011	0.035	0.75	0.045	−0.024	0.039
5.Saint Joseph’s	0.47	0.049	−0.024	0.043	0.53	0.051	−0.024	0.045
6.South Fork	0.23	0.051	−0.004	0.051	0.60	0.045	0.004	0.045
7.Reynolds Creek	0.85	0.024	−0.014	0.019	0.82	0.019	−0.006	0.018
8.Pali	0.56	0.025	0.017	0.019	0.80	0.022	0.017	0.013
9.Naqu	0.57	0.060	0.015	0.058	0.72	0.061	0.040	0.047
10.Yanco	0.41	0.053	−0.001	0.053	0.51	0.050	0.002	0.050
11.Kyeamba	0.36	0.077	−0.037	0.067	0.60	0.056	−0.010	0.055
12.REMEDHUS	0.55	0.047	0.021	0.042	0.67	0.035	0.015	0.032
13.Benin(2010–2014)	—	—	—	—	—	—	—	—
14.Niger(2010–2014)	—	—	—	—	—	—	—	—
Median	0.57	0.044	−0.003	0.040	0.72	0.045	−0.003	0.041
Flux Sites(Median)	0.23	0.062	0.013	0.056	0.42	0.059	0.009	0.052

Figure 4 shows time series comparison of the SMAPsm (red dots), NNsm-FY (blue dots) and in situ soil moisture observations (obs-sm in gray lines). For demonstration, we only show time series for one in situ network in every continent. NNsm-FY can reconstruct the pattern of SMAPsm including magnitude and variability, with a mean CC 0.86. Both NNsm-FY and SMAPsm show good agreement with in situ SSM (obs-sm) at daily time scale, capturing the daily and seasonal dynamics of SSM. For the Little Washita network, there is a slight underestimation during dry period. In Naqu, NNsm-FY has more retrievals than SMAPsm, especially in winter. Over Yanco, in many instances, NNsm-FY overestimates soil moisture associated with precipitation events, together with SMAPsm. For the AMMA-Benin, NNsm-FY overestimates soil moisture at both dry and wet period.

**Fig. 5:**
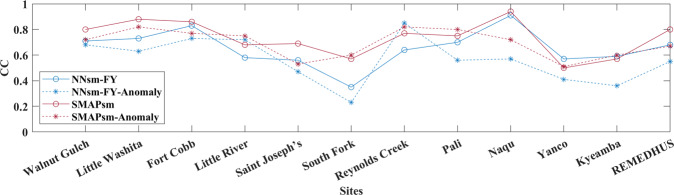
CC of NNsm-FY and SMAPL3sm against in situ soil moisture at 12 dense validation networks, at an independent validation period (2018–2019), for both actual SSM and SSM anomalies.

**Fig. 6:**
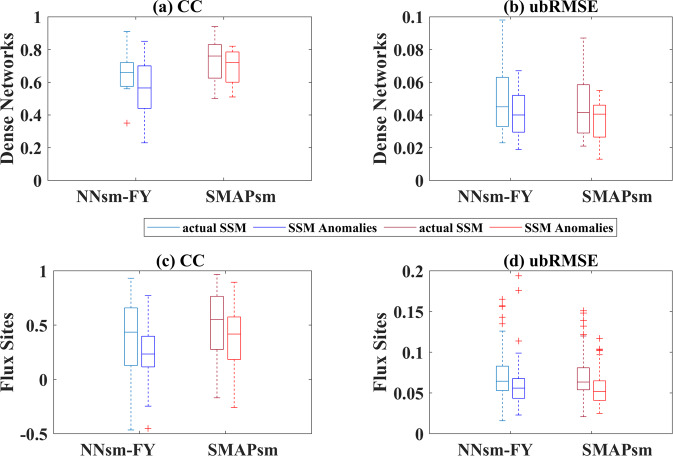
Box plots of statistics of NNsm-FY and SMAPsm against in situ soil moisture at an independent validation period (2018–2019), for both actual SSM and SSM anomalies: (**a**) CC, (**b**) ubRMSE(m^3^/m^3^) at dense networks, (**c**) CC and (**d**) ubRMSE(m^3^/m^3^) at flux sites.

Table 4 and plots in Figs. 5, 6 show statistical results for NNsm-FY and SMAPsm at dense networks and flux sites, at an independent validation period of 2018–2019, for both actual satellite SSM and SSM anomalies. For actual SSM and its anomalies, NNsm-FY generally have a lower accuracy than SMAPsm for most networks, with lower CC and higher ubRMSE. For actual SSM, correlations of NNsm-FY with in situ SSM are slightly lower than that of SMAPsm at dense netwoks, ranging from 0.56 to 0.91 (except 0.35 at South Fork) for NNsm-FY, and ranging from 0.50 to 0.94 for SMAPsm. For SSM anomalies, NNsm-FY performs worse than SMAPsm at most networks in terms of CC. For both NNsm-FY and SMAPsm, SSM anomalies have higher accuracy in terms of ubRMSE. Although the SSM anomalies show lower CC than actual SSM, both products still have relatively strong correlations and high accuracy after removing seasonal cycle.

From Fig. 5 over most sites CC of SSM anomalies are stable or slightly decrease from the CC of actual SSM, both for NNsm-FY and SMAP. Over site South Fork, NNsm-FY performs worse than SMAPsm, and it is more pronounced for SSM anomalies. Over site Naqu and site Kyeamba, NNsm-FY and SMAPsm have similar performance, but CC of NNsm-FY (Anomalies) is lower than CC of SMAP (Anomalies). There are several reasons for the difference of performance between NNsm-FY and SMAPsm. L-band on SMAP has higher sensitivity to soil moisture than high-frequency bands such as X-band,Ku-band and K-band on MWRI/FY-3B; The incident angle of SMAP (40°) is different with incident angle of MWRI/FY-3B (53°). Studies have shown that performance of soil moisture estimations is better at intermediate incidence angle of 40°to 45°, while performance will be degraded when incident angle is larger than 50°or less than 30°. And the sensitivity of different microwave bands to soil moisture and soil moisture change varies with the rainfall event, change of surface cover type and vegetation cover^51,52^.

### Validation and Comparison with FY-3B official product FY-sm at dense networks and flux sites

To further illustrate the accuracy of our product, we compared NNsm-FY with FY-3B official soil moisture product^53,54^ (FY-sm), at dense networks and flux sites.

**Fig. 7:**
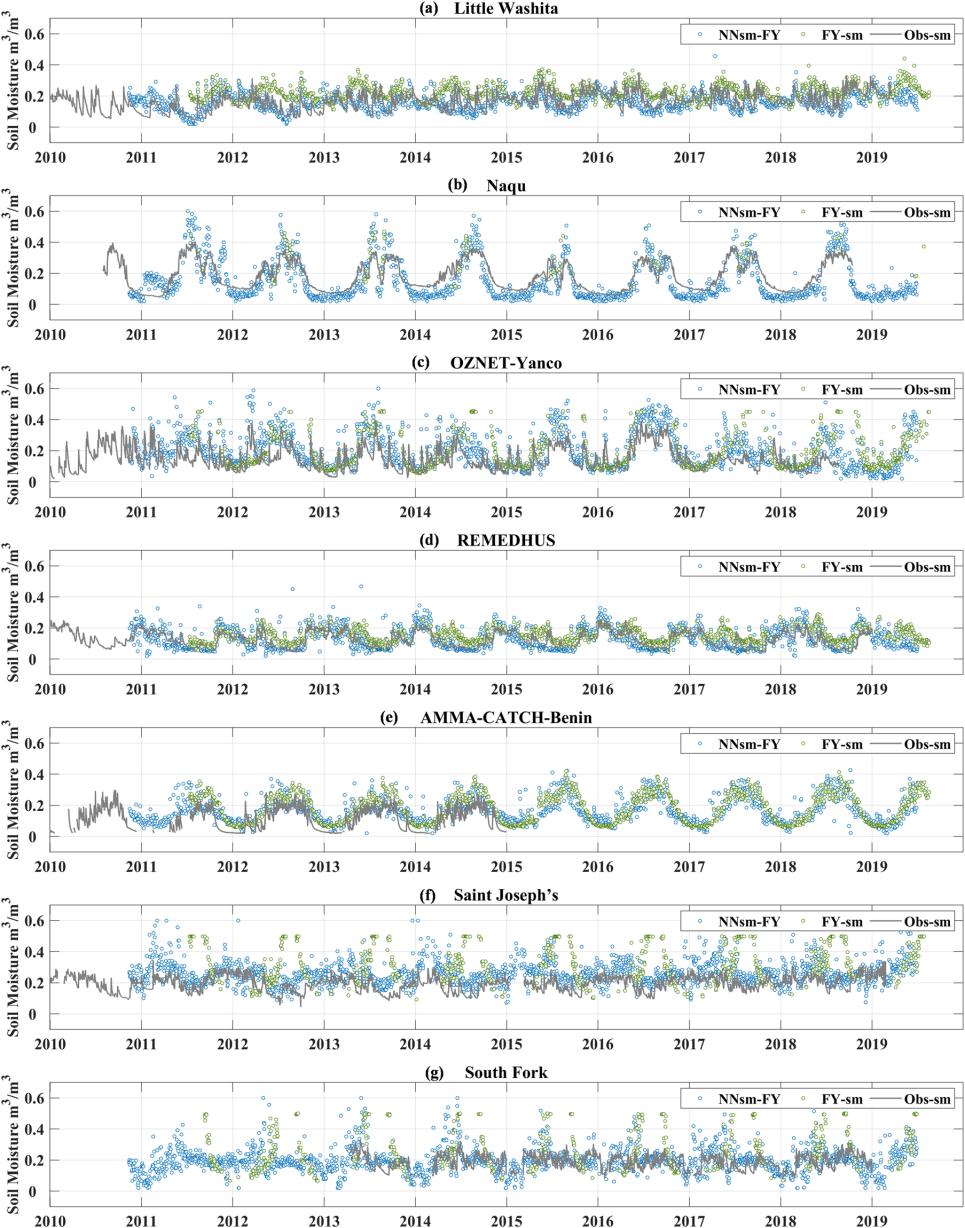
time series comparison of the NNsm-FY (blue dots), FY-sm (green dots) and in situ soil moisture observations (obs-sm in gray lines) for period 2010 to 2019 over in situ networks: (**a**) Little Washita, (**b**) Naqu, (**c**) Yanco, (**d**) REMEDHUS, (**e**) Benin, (**f**) Saint Joseph’s and (**g**) South Fork.

Figure 7 shows time series of the NNsm-FY (blue dots), FY-sm (green dots) and in situ soil moisture observations (obs-sm in gray lines) at dense networks. NNsm-FY shows good agreement with in situ observations, while FY-sm shows overestimation over some networks. For Little Washita network and REMEDHUS network, FY-sm significantly overestimates soil moisture. In Naqu network, FY-sm has very few retrievals only in Northern summer, and at Yanco network, FY-sm retrievals has very few retrievals in southern winter. For the AMMA-Benin, NNsm-FY and FY-sm have similar soil moisture distribution curve, and both products overestimates soil moisture, especially in dry period.

Table 5 shows statistical results for NNsm-FY and FY-sm at dense networks and flux sites for the whole FY-3B period (2010–2019). In terms of accuracy, our product NNsm-FY outperforms FY-sm, with lower ubRMSE and relatively higher CC (except Benin network). At Little River, Saint Joseph’s and South Fork networks, FY-sm highly overestimates soil moisture. FY-sm has no retrievals in northern winter caused by failure of algorithm and overestimate soil moisture in northern summer (as shown in the time series figures in Fig. 7f,g) at Saint Joseph’s and South Fork networks, which belong to the cold climate area according to Table 2. At those two networks, FY-sm even has no correlations with in situ observations. The same issues occur at two networks on the Tibetan Plateau, FY-sm has a few retrievals in northern summer at Naqu network, and has little retrievals at Pali networks.

**Table 5:** Statistical comparisons of NNsm-FY and FY-sm with in situ soil moisture for the whole FY-3B period (2010–2019).

2010–2019	NNsm-FY vs. in situ				FY-sm vs. in situ			
	CC	RMSE	Bias	ubRMSE	CC	RMSE	Bias	ubRMSE
1.Walnut Gulch	0.77	0.026	0.002	0.027	0.62	0.049	0.041	0.027
2.Little Washita	0.66	0.043	−0.007	0.043	0.41	0.081	0.061	0.054
3.Fort Cobb	0.72	0.047	−0.016	0.044	0.56	0.085	0.068	0.051
4.Little River	0.65	0.083	0.070	0.044	0.07	0.176	0.165	0.066
5.Saint Joseph’s	0.59	0.089	0.075	0.048	−0.06	0.195	0.148	0.120
6.South Fork	0.43	0.068	0.028	0.061	0.15	0.154	0.091	0.124
7.Reynolds Creek	0.63	0.058	−0.026	0.051	0.24	0.065	0.008	0.065
8.Pali	—	—	—	—	—	—	—	—
9.Naqu	0.70	0.071	0.017	0.069	0.70	0.087	0.050	0.071
10.Yanco	0.66	0.082	0.041	0.071	0.44	0.105	0.045	0.094
11.Kyeamba	0.57	0.125	0.067	0.105	0.53	0.168	0.130	0.107
12.REMEDHUS	0.76	0.040	0.007	0.040	0.64	0.054	0.039	0.037
13.Benin	0.78	0.073	0.057	0.046	0.88	0.086	0.076	0.041
14.Niger	0.52	0.029	0.018	0.023	0.40	0.047	0.039	0.026
Median	0.66	0.068	0.018	0.046	0.44	0.086	0.061	0.065
Flux sites(Median)	0.40	0.093	0.007	0.064	0.13	0.145	0.061	0.093
**2010–2019**	**NNsm-FY vs**. **in situ** **(Anomalies)**				**FY-sm vs**. **in situ** **(Anomalies)**			
1.Walnut Gulch	0.63	0.022	−0.001	0.022	0.54	0.019	−0.001	0.019
2.Little Washita	0.61	0.040	−0.005	0.040	0.60	0.039	−0.005	0.039
3.Fort Cobb	0.67	0.043	−0.005	0.043	0.62	0.040	−0.002	0.040
4.Little River	0.52	0.039	−0.001	0.039	0.50	0.035	0	0.035
5.Saint Joseph’s	0.53	0.048	−0.002	0.048	0.45	0.046	0.002	0.046
6.South Fork	0.50	0.056	0.006	0.056	0.54	0.044	0.014	0.042
7.Reynolds Creek	0.37	0.034	0.000	0.034	0.39	0.033	0	0.033
8.Pali	—	—	—	—	—	—	—	—
9.Naqu	0.40	0.079	−0.005	0.079	0.43	0.063	0.031	0.056
10.Yanco	0.54	0.064	0.001	0.064	0.39	0.052	0.006	0.051
11.Kyeamba	0.30	0.080	0.002	0.080	0.23	0.060	0.013	0.059
12.REMEDHUS	0.52	0.036	0.002	0.036	0.45	0.028	0.002	0.028
13.Benin	0.36	0.041	−0.007	0.041	0.28	0.029	−0.003	0.029
14.Niger	0.30	0.023	0.005	0.023	0.36	0.022	−0.004	0.021
Median	0.52	0.041	−0.001	0.041	0.47	0.039	0	0.039
Flux Sites(Median)	0.26	0.055	−0.001	0.054	0.22	0.050	0.001	0.048

**Fig. 8:**
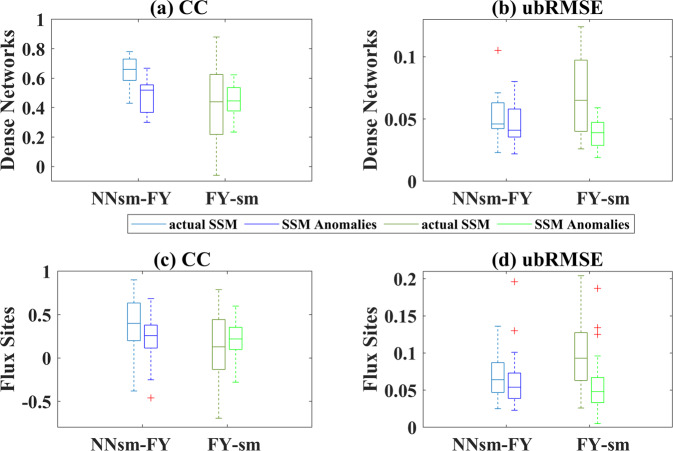
Box plots of statistics for NNsm-FY and FY-sm against in situ soil moisture observations for both actual SSM and SSM anomalies: (**a**) CC, (**b**) ubRMSE(m^3^/m^3^) at 13 dense validation networks, (**c**) CC and (**d**) ubRMSE(m^3^/m^3^) at flux sites.

Figure 8 shows the validation results at dense networks and flux sites. At dense networks, it is found that NNsm-FY has a relatively good performance with median CC of 0.66 and median ubRMSE of 0.046 m^3^/m^3^, while FY-sm has a worse performance with median CC of 0.44 and median ubRMSE of 0.065 m^3^/m^3^. At flux sites, although all statistical results get worse, NNsm-FY performs better than FY-sm. To be noted, there is a spatial scale mismatch between satellite and in situ flux sites soil moisture, as Cosh *et al*.^43^ demonstrated that at least 6 soil moisture sampling sites are necessary to adequately represent a 25 km × 25 km footprint-scale soil moisture. Here, soil moisture from flux sites, which is a measurement at point scale, is compared with 36 km × 36 km or 25 km × 25 km averaged satellite observation. These statistical results have certain uncertainty and are found lower than that from dense networks.

### Comparison with ERA5 soil moisture

**Fig. 9:**
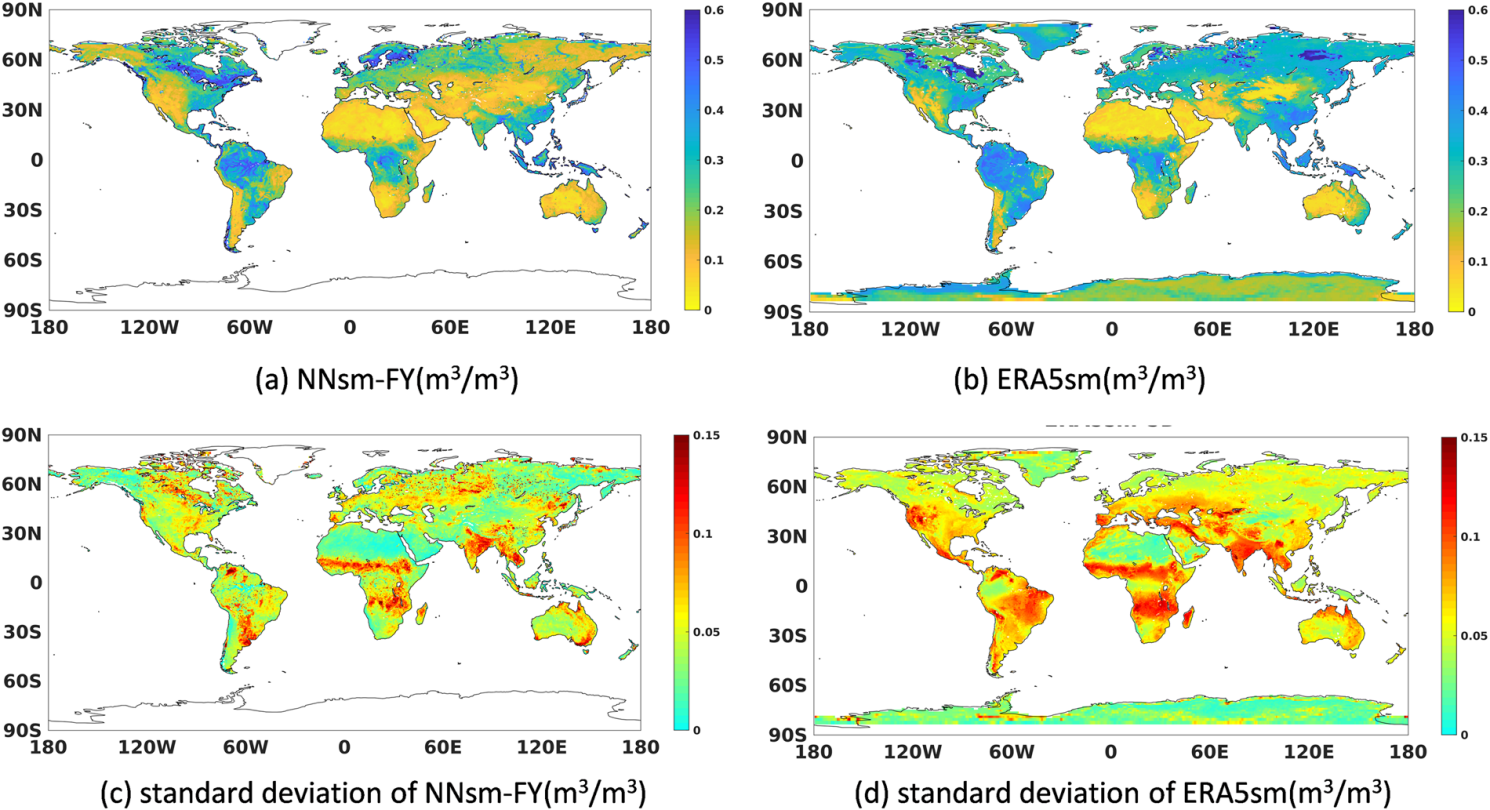
Global distribution of daily averaged soil moisture and standard deviation from 2010 to 2019.

**Fig. 10:**
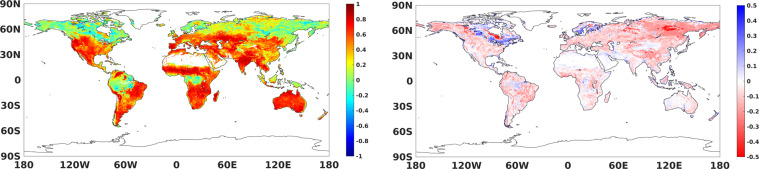
Comparisons of daily data of NNsm-FY with ERA5sm in the whole FY-3B period (2010–2019) at each grid cell globally: (**a**) CC, (**b**) Bias(m^3^/m^3^).

Due to the limited coverage of ground soil moisture observations, we complemented the comparison between our product and ERA5 soil moisture (ERA5sm, first layer soil moisture of fifth generation of European Centre for Medium Range Weather Forecasts (ECMWF) reanalysis data). We presented the global distribution of mean and standard deviation values of NNsm-FY and ERA5sm in Fig. 9, and the statistical results of CC and bias between the two in Fig. 10.

In Fig. 9a,b, at a global scale, NNsm-FY shows very similar spatial pattern as ERA5sm but it’s globally drier than ERA5sm, with lower SSM in Sahara, Australia and other arid areas, and higher SSM in tropical rainforests and in northern high latitudes. There are two exceptions to this similar pattern, the eastern Russia, and the Great Lakes and adjacent coniferous forest area of Canada. In eastern Russia, NNsm-FY is relatively dry, while ERA5sm is wetter. In the Great Lakes and adjacent coniferous forest area of Canada, NNsm-FY is wetter than ERA5sm. These two exceptions are also displayed in the bias map in Fig. 10b. The standard deviation (SD) of soil moisture in Fig. 9c,d is calculated on the daily soil moisture from 2010 to 2019. SD is an excellent descriptor of the average seasonal variation. SD is low for long-term dry and wet areas, indicating less variability in soil moisture for those areas such as tropical regions and deserts. Both NNsm-FY and ERA5sm have a high seasonal variation (high SD values) in the Sahel region, the southern part of Central and East Africa, India. SD of NNsm-FY are systematically lower than SD of ERA5sm, indicating that ERA5sm may overestimate SSM variability, which was also evidenced in researches when comparing ERA5sm with SMAPsm or in situ data from agrometeorological stations^55,56^.

Figure 10 shows the statistical results of CC and Bias between daily data of NNsm-FY with ERA5sm in the whole FY-3B period from 2010 to 2019. In general correlations are positive over most regions globally, and relatively strong correlations occur in regions with high seasonal variability in soil moisture, where may lack of seasonal repeatability. This indicates that the neural network is indeed working. It is reasonable that the correlation can be very weak for regions with lower values of SD of soil moisture.

### Homogeneity of NNsm_FY and NNsm_AMSR

1. Fill in gap of NNsm-AMSR
  Previously developed product NNsm-AMSR^39^ has a similar accuracy with SMAPL3sm, successfully transferring high accuracy of L-band SMAP to C/X-band AMSR. Unfortunately, NNsm-AMSR has a gap in period from Oct. 2011 to Jun 2012, because AMSR-E and its successive sensor AMSR2 have a gap in this period. This gap limits application of NNsm-AMSR such as drought analysis and climate change research. NNsm-FY dataset developed in this research, spanning from late 2010 to 2019, exactly fill in this gap, with a similar high accuracy with SMAP, as shown in the red dotted box in Fig. 11.

**Fig. 11:**
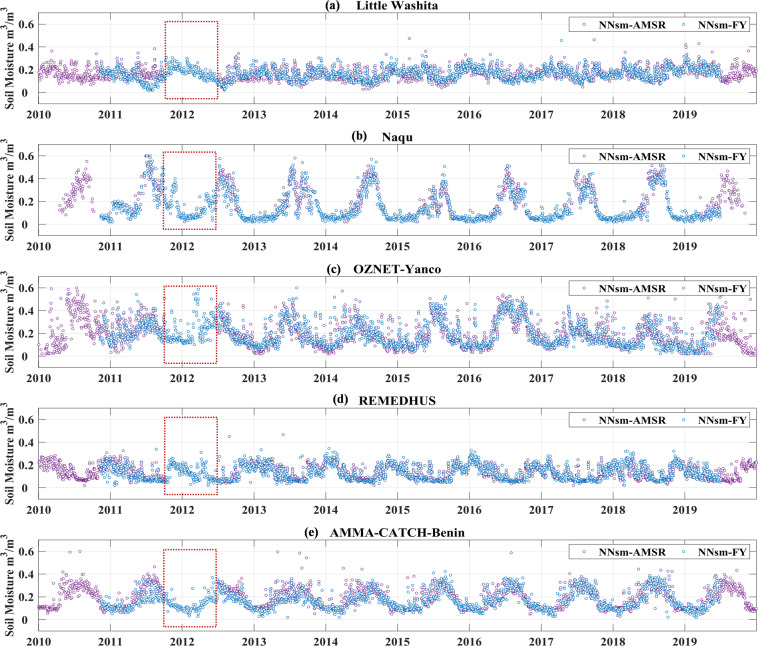
time series comparison of the NNsm-AMSR (purple dots) and NNsm-FY (blue dots) for period 2010 to 2019 over in situ networks: (**a**) Little Washita, (**b**) Naqu, (**c**) Yanco, (**d**) REMEDHUS, (**e**) Benin.
2. Homogeneity of NNsm_FY and NNsm_AMSR

**Fig. 12:**
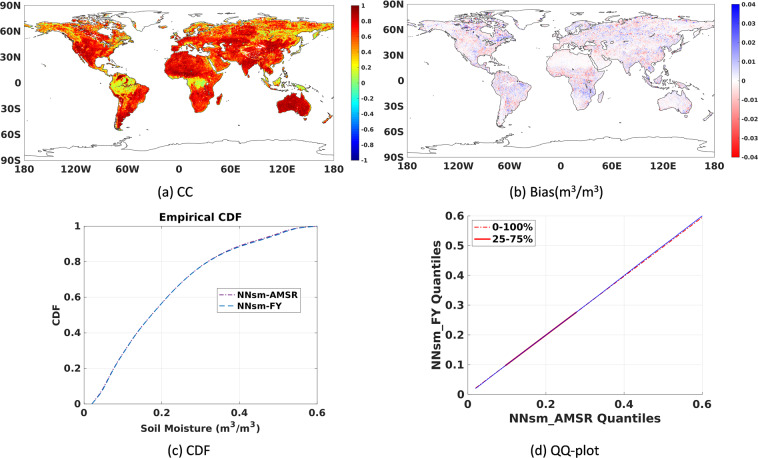
Statistical comparisons of NNsm-FY with NNsm-AMSR in the whole FY-3B period (2010–2019) at each grid cell globally: (**a**) CC, (**b**) Bias(m^3^/m^3^), (**c**)CDF and (**d**) Q-Q plot.

Before combination usage of NNsm_FY and NNsm_AMSR, we evaluated the homogeneity of those two datasets. As shown in Fig. 11, NNsm_FY and NNsm_AMSR have the similar dynamic range and values of soil moisture. We also tested the consistency in terms of CC, bias Cumulative Distribution Function (CDF), and Quantile-Quantile plot(Q-Q plot), shown in the Fig. 12 (all global grid cells) and Table 6 (taking the grid cells where validation networks located as examples). The Q-Q plot and CDF, based on all of the daily data for the NNsm_FY and NNsm_AMSR in Fig. 12c,d, demonstrates that those two datasets have similar spatio-temporal distribution pattern. NNsm_FY agrees well with NNsm_AMSR, with high CC values in regions with significant soil moisture dynamics, and with most absolute values of bias less than 0.01 m^3^/m^3^, as shown in the Fig. 12a,b and Table 6.

**Table 6:** Statistical comparisons of NNsm-FY with NNsm-AMSR in the whole FY-3B period (2010–2019) at grid cells where 14 dense validation networks located.

Locations of grid cells	NNsm-FY vs. NNsm-AMSR	
	CC	Bias
1.Walnut Gulch	0.82	0.0002
2.Little Washita	0.76	−0.001
3.Fort Cobb	0.79	0.017
4.Little River	0.84	0.005
5.Saint Joseph’s	0.72	0.002
6.South Fork	0.80	0.014
7.Reynolds Creek	0.75	−0.006
8.Pali	0.77	0.006
9.Naqu	0.90	−0.140
10.Yanco	0.85	0.005
11.Kyeamba	0.76	−0.002
12.REMEDHUS	0.83	0.001
13.Benin	0.85	−0.015
14.Niger	0.75	−0.003

### Advantages of NNsm-FY

As described in the previous section, NNsm-FY has high accuracy, showing good agreement with SMAPsm and in situ observations, and performs better than FY-3B official soil moisture product (FY-sm) when comparing with in situ soil moisture. Filling in gap of NNsm-AMSR (2011.11–2012.06), NNsm-FY works together with NNsm-AMSR to provide complete long-term soil moisture since 2002, both having a high accuracy similar with SMAPsm.

**Fig. 13:**
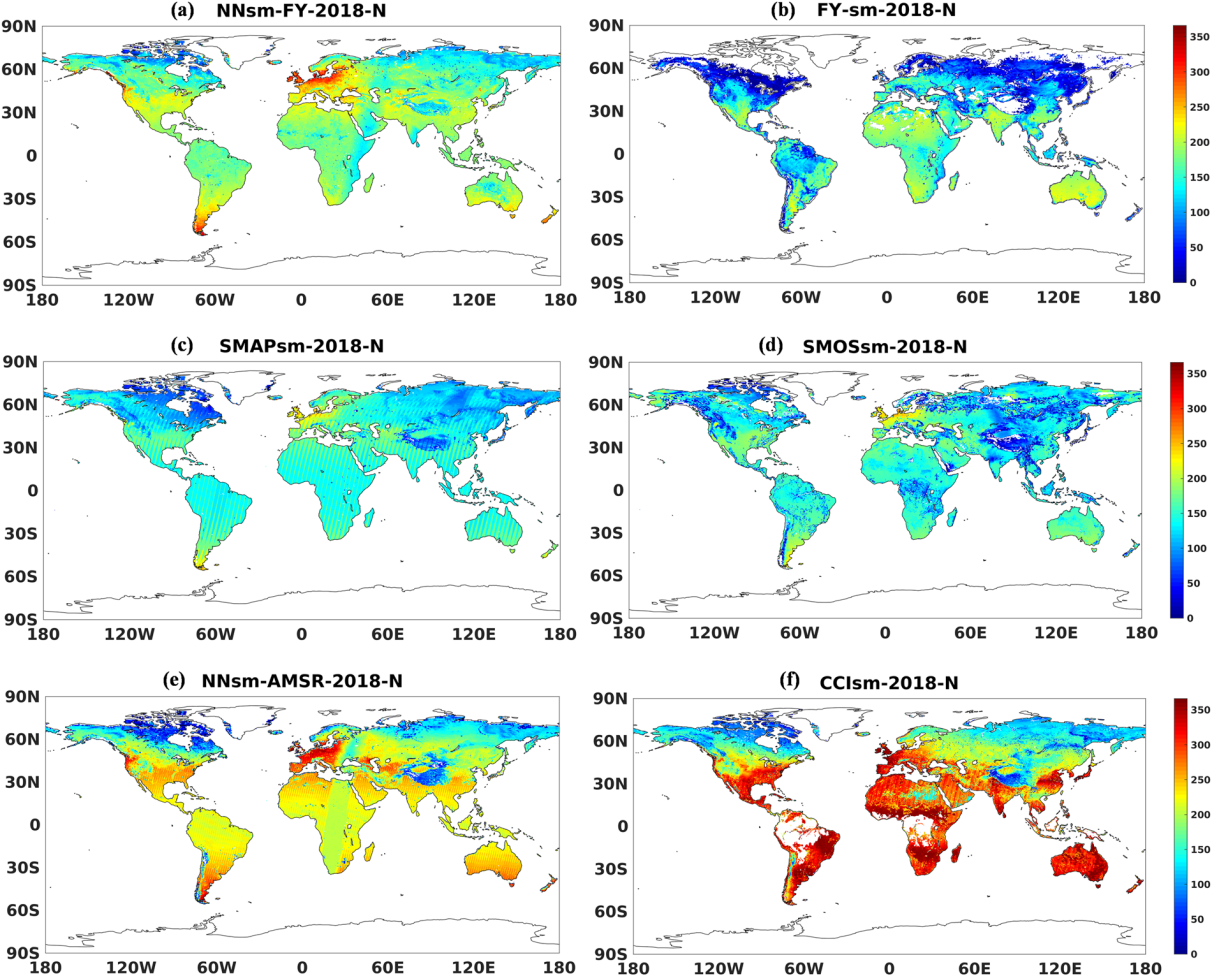
Amount of soil moisture retrievals at each grid cell within 2018 for the following products: (**a**) NNsm-FY, (**b**) FY-sm, (**c**) SMAPsm, (**d**) SMOSsm, (**e**) NNsm-AMSR, and (**f**) CCIsm.

For the convenience of application, we also compare data spatial coverage, taking 2018 as an example. In Fig. 13a, NNsm-FY can provide considerable amount of soil moisture retrievals at each grid cell globally. Another important detail worth mentioning is that, NNsm-FY has more soil moisture retrievals in the Tibetan Plateau and in some part of the high latitudes. FY-sm shown in Fig. 13b has no retrievals at most grid cell in the Tibetan Plateau, and has less retrievals in the high latitudes, caused by the failure of algorithm or flagging of frozen soil. In addition to high accuracy, our product NNsm-FY has a greater spatial coverage than SMAPsm and SMOSsm, especially in the Tibetan Plateau area, as shown in Fig. 13a,c,d. This is primarily determined by the satellite configuration, as swath width of SMAP and SMOS is 1000 km and swath width of FY-3 MWRI is 1400 km. In the Tibetan Plateau area, NNsm-FY has more retrievals both for swath width and retrieval algorithm. NNsm-AMSR in Fig. 13e can provide most soil moisture retrievals globally among those 5 single satellite products, except that NNsm-FY has more retrievals than NNsm-AMSR in the Tibetan Plateau. In Fig. 13f for CCIsm(Version v05.2), numbers of retrievals per year at some medium- and low-latitude grid cells are close to or equal to 365, namely CCIsm has retrievals almost every day at those gird cells, which is because CCIsm merged ASCAT, AMSR2, SMOS and SMAP into one product^57^. Even so, CCIsm has less retrievals in the Tibetan Plateau (less than 100 retrievals per year at one grid cell), due to the LPRM retrieval algorithm as well as different quality and uncertainties of other used soil moisture products.

## Data Availability

The input data processing, dataset generation and validation were conducted using Matlab software (R2018b version). Code is available on Github: https://github.com/panpanyao/NNsm-FY-code.
